# An Improved Bird Detection Method Using Surveillance Videos from Poyang Lake Based on YOLOv8

**DOI:** 10.3390/ani14233353

**Published:** 2024-11-21

**Authors:** Jianchao Ma, Jiayuan Guo, Xiaolong Zheng, Chaoyang Fang

**Affiliations:** 1 School of Geography and Environment, Jiangxi Normal University, Nanchang 330022, China; 202240100109@jxnu.edu.cn; 2 Key Laboratory of Poyang Lake Wetland and Watershed Research, Ministry of Education, Jiangxi Normal University, Nanchang 330022, China; 3 Jiangxi Ziwu Universal Information Technology Co., Ltd., Nanchang 330095, China; guojiayuan043@gmail.com (J.G.); zhengxlqsol@gmail.com (X.Z.)

**Keywords:** deep learning, attention mechanism, small-object-detection layer, lightweight detection head, loss function

## Abstract

Poyang Lake is the largest freshwater lake in China and plays a significant ecological role. Deep-learning-based video surveillance can effectively monitor bird species on the lake, contributing to the local biodiversity preservation. To address the challenges of multi-scale object detection against complex backgrounds, such as a high density and severe occlusion, we propose a new model known as the YOLOv8-bird model. First, we use Receptive-Field Attention convolution, which improves the model’s ability to capture and utilize image information. Second, we redesign a feature fusion network, termed the DyASF-P2, which enhances the network’s ability to capture small object features and reduces the target information loss. Third, a lightweight detection head is designed to effectively reduce the model’s size without sacrificing the precision. Last, the Inner-ShapeIoU loss function is proposed to address the multi-scale bird localization challenge. Experimental results on the PYL-5-2023 dataset demonstrate that the YOLOv8-bird model achieves precision, recall, mAP@0.5, and mAP@0.5:0.95 scores of 94.6%, 89.4%, 94.8%, and 70.4%, respectively. Additionally, the model outperforms other mainstream object detection models in terms of accuracy. These results indicate that the proposed YOLOv8-bird model is well-suited for bird detection and counting tasks, which enable it to support biodiversity monitoring in the complex environment of Poyang Lake.

## Simple Summary

Monitoring endangered bird species is critical for the conservation and management of wetland ecosystems. This monitoring can be carried out in real-time by integrating deep learning with video surveillance technology. This study describes an improved bird detection method by adding several new modules to the YOLOv8 model, including a Receptive-Field Attention convolution (RFAConv), a DyASF-P2 feature fusion network consisting of a DySample, a Scale Sequence Feature Fusion (SSFF) module, a Triple Feature Encoding (TFE) module, and a small-object-detection layer. Other modules include a lightweight detection head and an Inner-Shape Intersection over Union (Inner-ShapeIoU) loss function. The resulting model is termed the YOLOv8-bird, which outperforms commonly used real-time detection networks. It offers both high accuracy and fast detection, making it well-suited for the identification of bird species in real-time video surveillance systems.

## 1. Introduction

Poyang Lake, China’s largest freshwater lake, plays essential roles in climate improvement, pollution control, and ensuring the regional ecological balance [1,2]. At the same time, it offers ideal conditions for rare and migratory birds. Every winter, thousands of water fowl migrate to the lake, enhancing its rich biodiversity [3]. However, human activities and global climate change have intensified the species extinction and ecosystem degradation process, threatening its ecological integrity and biodiversity. Birds, as key indicators of the ecosystem health [4], can provide valuable insights into the composition and size of bird communities, which may be gained by conducting bird counting. This information is crucial for evaluating the health of wetland environments and assessing bird diversity. Bird recognition plays a crucial role in bird monitoring, ecological conservation, and biodiversity research, and its automation enables researchers to efficiently classify and detect bird species across various regions. Given the location’s significance, new techniques and a systematic monitoring system should be urgently developed to improve the long-term observation of bird diversity in the Poyang Lake wetland.

Traditional and widely utilized bird diversity monitoring methods include line transects, catching and marking, and point counts [5,6,7]. They offer insights into the bird community structure, population, and spatial distribution in wetlands. However, these methods have notable drawbacks, including high human and material resource consumption, a limited monitoring frequency and coverage, weather sensitivity, and dependence on the observer’s experience.

Recent advancements in automatic remote monitoring devices and deep learning have enabled continuous bird monitoring. This approach minimizes the human disturbance, providing all-weather and year-round data collection that yields more accurate information on the bird richness and distribution. The collected data can be categorized into audio and video formats. Some researchers [8,9,10] carried out feature extraction for sound recognition by utilizing spectrograms as inputs of convolutional neural networks, ultimately achieving favorable bird song recognition results. Szegedy et al. [11] introduced the Inception neural network architecture to obtain more comprehensive information compared to the single-kernel convolution layer architecture. Additionally, some studies [12,13] gradually integrated attention mechanisms into bird sound classification networks to enhance the ability to capture long-range dependencies in feature maps. However, bird song classification has significant limitations compared to video data classification. Simultaneous vocalizations from multiple bird species make it complicated to distinguish between individual calls. Furthermore, audio feature extraction becomes challenging due to the time-dependent nature of a bird song and its complex frequency and rhythm. In contrast, visual features are not time-dependent, which makes it easier to use them for the analysis of bird morphology and behavior from static images. Some of the existing work [14,15,16] has employed various object-detection framework algorithms for bird recognition and comparison, validating the usefulness of the integration of automatic remote detection devices with deep learning for bird detection across diverse environments. Lei et al. [17] incorporated additional prediction heads and a simple and parameter-free attention module into the You Only Look Once v7, model to enhance the bird recognition accuracy from images. Wu et al. [18] developed a two-step framework for waterbird monitoring, involving (1) video segmentation and stitching into panoramic images; and (2) counting and density estimation using a depth density estimation network.

In terms of bird recognition in images, most existing studies concentrate on identifying specific bird species using fine-grained information, which limits their effectiveness for real-time counting and monitoring. Different challenges include the following: (1) The complex Poyang Lake environment causes redundant background information to appear in images, which complicates feature extraction. (2) Smaller birds are detected with a low accuracy that is exacerbated by their high density and occlusion. (3) Multi-scale variations make it difficult to localize the targets.

The aforementioned challenges lead to false positives and missed detections, which reduce the detection accuracy and complicate the precise bird counting process. Additionally, the detection accuracy and speed must be balanced for effective real-time monitoring in video surveillance applications. To address these challenges, this study constructed a dataset comprising 8077 images tailored for the bird counting task on Poyang Lake. Furthermore, an improved YOLOv8 model, termed the YOLOv8-bird model, is proposed to identify rare bird species in the Poyang Lake wetland environment. The improvements in this model include the following components: (1) Receptive-Field Attention convolution is used in the model, which allows it to dynamically allocate receptive field weights based on the importance of input features. Consequently, the model’s ability to capture fine-grained details of birds is improved while the background noise is suppressed. (2) The improved DyASF-P2 feature fusion module [19,20] is utilized to enhance the model’s ability to extract multi-scale contextual and global information, improving the recognition of dense and small targets and enabling accurate bird counting in high-density scenes. (3) A lightweight detection head is introduced, which increases the detection speed of the network while maintaining its accuracy. (4) The Inner-ShapeIoU loss function is proposed, which exploits the scale-aware advantages of the Shape Intersection over Union (Shape-IoU) [21] and incorporates the idea of auxiliary bounding boxes from the Inner Intersection over Union (Inner-IoU) [22] to accelerate convergence. This improved loss function effectively addresses the challenges of multi-scale object localization.

The improved algorithm can be deployed on existing surveillance cameras to monitor birds with a high accuracy and potentially contribute to wildlife conservation efforts.

## 2. Materials and Methods

### 2.1. Bird Dataset

**Figure 1:**
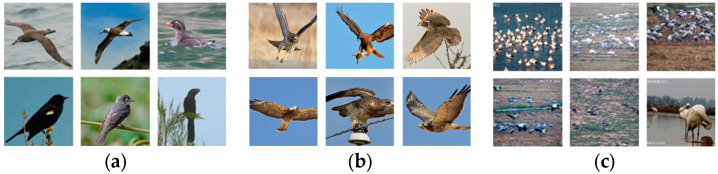
Comparison of datasets: (**a**) Caltech-UCSD Birds-200-2011; (**b**) North America Birds; and (**c**) PYL-5-2023.

The bird identification accuracy and reliability depend heavily on the availability of rich and diverse datasets. This is especially important due to the dynamic behavior of birds, such as flight and perching postures, as well as their activities in different environments, e.g., lighting conditions, background complexity, and occlusion, all of which affect the recognition accuracy. Commonly used bird detection datasets which focus on fine-grained bird identification like Caltech-UCSD Birds-200-2011 [23] and North America Birds [24] typically include only one bird per image and do not contain the complex background conditions, occlusions (within species, between different species, and from the environment), and multi-scale targets encountered in real-world scenarios (Figure 1). As such, they may not be useable for bird recognition tasks in natural settings.

To address these limitations, we collected and prepared the Poyang Lake Bird Dataset (PYL-5-2023). The dataset is derived from historical surveillance footage provided by the “Poyang Lake Wetland Ecosystem Monitoring and Early Warning Platform”. The cameras used for surveillance are Dahua DH-PTZ-85260-HNF-WHC-E integrated PTZ cameras, which capture videos at a frame rate of 25 frames per second. The detailed information of the PTZ camera is shown in Figure S1 and Table S1. The video data span from 1 February 2022 to 10 January 2024 and include the monitoring sites of Wuxing Farm, East Poyang Lake, and Yugan. Images are collected from the video footage under various lighting conditions, postures, and occlusions for both single-target and multi-target bird scenarios. Blurry images caused by weather conditions or camera issues are manually removed, resulting in 8077 high-quality bird images.

In this study, we focus on five typical bird species of Poyang Lake: the Siberian White Crane (*Grus leucogeranus*), Greater White-fronted Goose (*Anser albifrons*), Oriental White Stork (*Ciconia boyciana*), Common Crane (*Grus grus*), and Tundra Swan (*Cygnus columbianus*). Bird targets are manually annotated using the X-AnyLabeling tool v2.0.0, where the annotations cover both bird attributes and positional information. The dataset is split into training (5653 images), validation (1616 images), and test sets (808 images) in a 7:2:1 ratio. A detailed display of the dataset is shown in Figure S2.

### 2.2. YOLOv8 Detection Network

Object detection is one of the core research areas in computer vision that uses algorithms to detect and localize specific target classes from images or videos [25]. The object detection algorithms can generally be divided into two-stage and single-stage detectors. The former include R-CNN [26], Faster R-CNN [27], Mask R-CNN [28], and Cascade R-CNN [29], which typically first use a Region Proposal Network to generate candidate regions, followed by a further refinement of classification and bounding boxes to generate precise detection results. These two-stage detectors offer higher accuracy at the cost of increased computational complexity. On the other hand, single-stage detectors like the You Only Look Once (YOLO) [30,31,32,33] series and Single Shot MultiBox Detector (SSD) [34] directly extract features to predict both object classification and location, which improves the detection speed but slightly deteriorates the accuracy.

**Figure 2:**
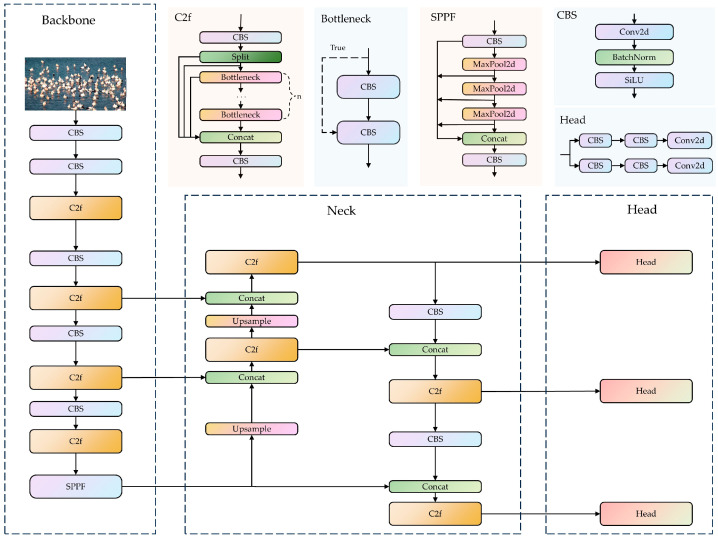
YOLOv8 network structure. (1) The YOLOv8 network model is composed of three parts: the backbone, the neck, and the head. (2) YOLOV8 includes CBS module, the Cross Stage Partial with two fusion (C2f) module, the Spatial Pyramid Pooling-Fast (SSFF) module, and Head module. The CBS module consists of convolution, batchnorm, and a sigmoid linear unit (SiLu) function, and it is named after the initials of these components. The Head module is used for the final classification and regression tasks. (3) The Concat is used to concatenate multiple data tensors along a specified dimension.

The YOLO algorithm is more suitable for balancing the detection accuracy and speed for real-time monitoring tasks on Poyang Lake compared to the existing algorithms. The YOLOv8 network model is composed of three main parts: the backbone, the neck, and the head (Figure 2). The backbone utilizes the Cross Stage Partial with two fusion (C2f) module, and the Spatial Pyramid Pooling-Fast (SPPF) module. The C2f module enhances the feature extraction accuracy by repeatedly splitting gradients and stacking many residual modules, and subsequently merging branches to obtain the enhanced features. The SPPF module performs three consecutive pooling and downsampling operations, which are used to integrate features from different scales to enrich the semantic content of the feature maps. The neck network employs a path aggregation network structure, which promotes feature fusion across objects of different scales. The classification and detection processes are decoupled in the head network, where the detection head carries out object localization and classification, ultimately generating the final object detection results. The classification and detection heads are separate, transitioning from a coupled structure to a more advanced decoupled head structure. Furthermore, an Anchor-Free [35] detection head is used in place of the Anchor-Based detection head, which reduces the number of anchor predictions and speeds up the Non-Maximum Suppression process.

### 2.3. Proposed YOLOv8-Bird Detection Network

**Figure 3:**
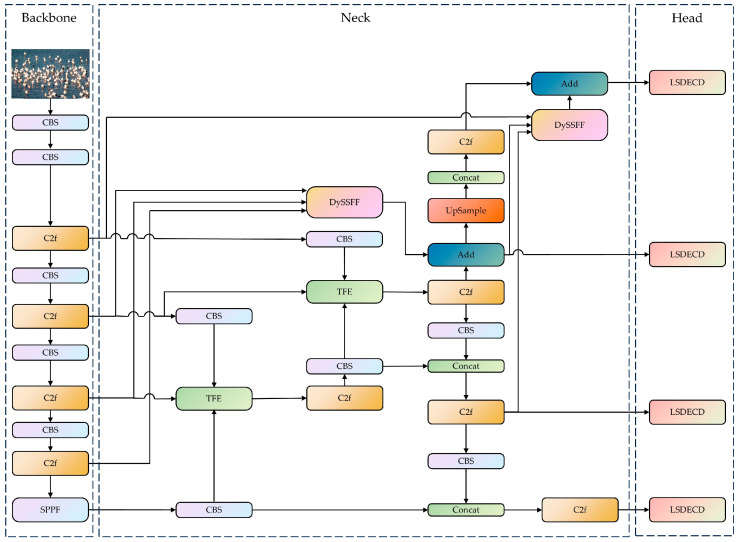
YOLOv8-bird network structure. (1) The YOLOv8-bird network model is composed of three parts: the backbone, the neck, and the head. (2) The YOLOV8-bird includes CBS module, the Cross Stage Partial with two fusion (C2f) module, the Spatial Pyramid Pooling-Fast (SSFF) module, the scale sequence feature fusion module combined with dynamic upsampling (DySSFF), a triple feature-encoding (TFE) module, and a lightweight, shared-detail-enhanced detection head (LSDECD). The CBS module consists of convolution, batchnorm, and a sigmoid linear unit (SiLu) function, and it is named after the initials of these components. (3) The concat is used to concatenate multiple data tensors along a specified dimension. The Add is an operation that overlays feature information of the same dimension.

In this paper, the YOLOv8-bird model, which uses YOLOv8 as the base model, is proposed for rare bird detection on Poyang Lake. The neck network containing the DySample, the SSFF modules, and TFE modules are designed by combining a small-target-detection layer. Furthermore, RFAConv; a lightweight, shared-detail-enhanced detection head; and Inner-ShapeIoU are introduced to enhance the model’s adaptive ability for input images of different sizes and to mitigate the problems of occluded target alignment error, local aliasing, and missing features (Figure 3).

#### 2.3.1. Improved Convolution Module

The environment where birds reside on Poyang Lake is highly diverse and complex, encompassing mountains, rivers, and sandy areas. Additionally, the birds exhibit varying postures with rich detail and features. However, the YOLOv8 model’s ability to learn intricate patterns is significantly limited as the standard convolution operations use shared parameters. Moreover, bird targets are often occluded by elements like branches and leaves, which introduce considerable noise that restricts the model’s performance. Standard spatial attention mechanisms may also be insufficient to capture all relevant information. RFAConv solves the problem of overlapping receptive field features in large kernel convolution operations of spatial attention mechanisms. To address these challenges, we replace the conventional convolution modules with RFAConv to reduce the background noise and enhance the network’s ability to perceive important features [36]. These changes improve the detection performance.

**Figure 4:**
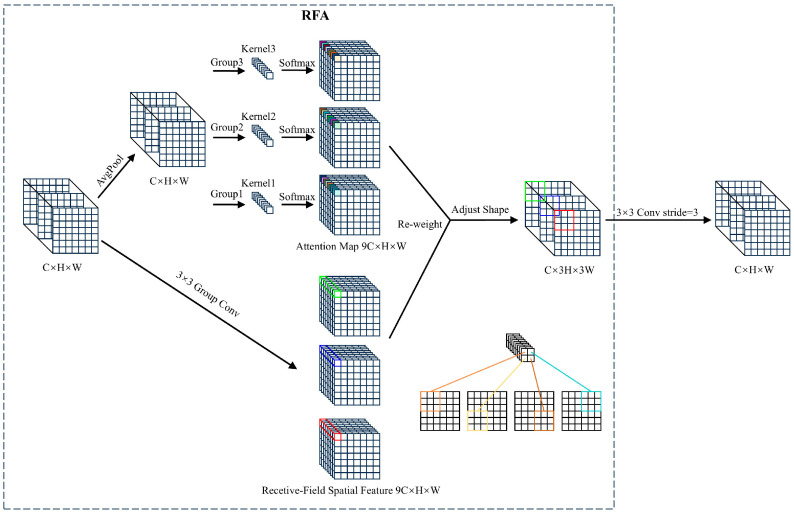
The overall structure of Receptive-Field Attention with a 3 × 3 kernel. C, H, and W denote the number of channels and the height and width of the feature map, respectively. The parameters are different between the group convolution’s kernels. Different colors represent different feature information extracted through different receptive fields. The 3 × 3 Group Conv represents the group convolution operation with a kernel size of 3 × 3. The AvgPool represents average pooling operation.

First, convolution is applied to the input feature map to generate an initial set of feature maps. The additional computational overhead associated with interactions between receptive field features is reduced by using average pooling to aggregate the global information for each receptive field. Second, a 1 × 1 group convolution is performed to facilitate information exchange. Last, Softmax is employed to emphasize the importance of each feature within the receptive field (Figure 4).

In general, Receptive-Field Attention (RFA) computation can be represented as follows:

(1) $$F=Softmax\left(g^{1\times 1}\left(AvgPool\left(X\right)\right)\right)\times ReLu\left(Norm\left(g^{k\times k}\left(X\right)\right)\right)=A_{rf}\times F_{rf}$$

where g^i×i^ denotes a group convolution of size i × i, k represents the kernel size, Norm refers to normalization, and X is the input feature map. Softmax represents the normalized exponential function. ReLu represents the rectified linear unit function. AvgPool represents average pooling. The output feature map F is obtained by the multiplication of the attention map A_rf_ with the transformed receptive-field features F_rf_. RFAConv considers the importance of features within each receptive field, addressing the limitations of standard convolutions caused by shared parameters and insensitivity to positional changes.

#### 2.3.2. Improved Feature Fusion Module

Feature fusion networks can integrate feature information from various levels, enabling the effective detection of multi-scale targets. Continuous downsampling and feature extraction are used to increase the semantic richness of the features while decreasing the noise levels. However, this causes a shrinking output feature map scale, due to which the downsampling results can exceed the size of small objects. Consequently, feature information may be partially lost in the transmitted feature map, which makes it nearly impossible for the subsequent top-down Feature Pyramid Network to integrate features of small objects. Additionally, deep feature maps acquire a greater amount of semantic information but lose spatial details with the increasing network depth, which affects the object detection accuracy. To solve this issue, we redesign the feature fusion network structure, which is made up of five components: an additional P2 detection layer specifically for small object features, two TFE modules, and two improved scale sequence feature fusion based on DySample (DySSFF) modules.

SSFF enhances the feature fusion ability by effectively integrating information from different feature maps. It leverages the scale-space characteristics of images, maintaining scale-invariant features during downsampling and extracting key information through upsampling. Subsequently, a Gaussian filter is applied to smooth the upsampled images and generate images of the same resolution but at different scales, and three-dimensional convolution is employed to extract their scale sequence features. The specific expression for image scaling is as follows:

(2) $$F_{\sigma }\left(w,h\right)=G_{\sigma }\left(w,h\right)\times f\left(w,h\right)$$

(3) $$G_{\sigma }\left(w,h\right)=\frac{1}{2\pi \sigma^{2}}e^{-\frac{w^{2}+h^{2}}{2\sigma^{2}}}$$

where f(w, h) represents a two-dimensional input image of width w and height h. The output Fσ(w, h) is generated by a series of smoothing convolutions using the two-dimensional Gaussian filter Gσ(w, h), whose standard deviation scaling parameter is denoted by σ.

**Figure 5:**
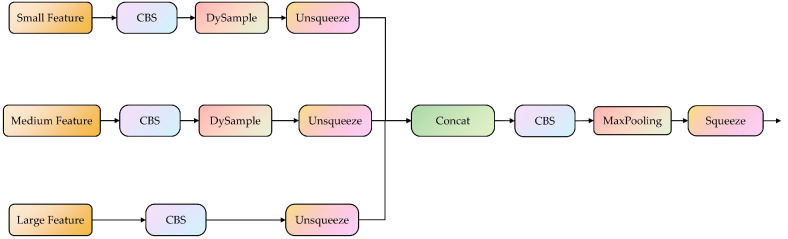
The overall structure of scale sequence feature fusion based on the dynamic sampling module. The CBS module consists of convolution, batchnorm, and a sigmoid linear unit function. The Unsqueeze and Squeeze are operations used to increase and dacrease the dimensions of data, respectively. The concat is used to concatenate multiple data tensors along a specified dimension.

The DySample module avoids the potential loss of small-object feature information during upsampling by specifying the sampling angles during upsampling [37], effectively exploiting semantic information within feature maps, and enriching the semantic relationships at different scales. Compared to traditional upsampling methods, DySample has a better adaptability to the pixel information and content features of the feature map. Figure 5 shows the improved DySSFF module structure. By combining DySample and the SSFF, the resulting DySSFF module fully leverages the advantages of dynamic upsampling and feature fusion. It enhances the model’s feature fusion capabilities, achieves more efficient multi-scale feature integration, and prevents the loss of small-object feature information.

Densely overlapping birds can be identified by expanding the image information to compare posture variations at different scales. This is achieved by introducing the TFE module, which splits, adjusts, and fuses large-, medium-, and small-scale features to enhance the detailed feature information. The input to the TFE consists of large-, medium-, and small-scale features (Figure S3). The number of feature channels is adjusted prior to feature encoding to align with the primary scale features. For large-scale feature maps, max pooling and average pooling are combined and used for downsampling and preserving high-resolution features and details. On the other hand, nearest-neighbor interpolation is applied for upsampling to maintain the richness of local features and prevent any information loss in small-scale feature maps. Last, the three feature maps of the same size are concatenated along the channel dimension. This step enables the network to capture multi-scale target information, improving its detection performance and enhancing the feature information. This process can be described as follows:

(4) $$F_{TFE}=Concat\left(F_{l},F_{m},F_{s}\right)$$

where *F_TFE_* represents the output feature map of the TFE module obtained by the concatenation of *F_l_*, *F_m_*, and *F_s_*, which represent the large-, medium-, and small-scale feature maps, respectively. Concat represents the concatenation of feature maps from different levels along a specific dimension.

The original YOLOv8 network uses three detection heads to process feature maps at different scales: 80 × 80, 40 × 40, and 20 × 20. Larger-resolution feature maps have smaller receptive fields and provide detailed local features and position information, which facilitate the recognition of small objects. In contrast, smaller feature maps have larger receptive fields and capture richer semantic information, which increases their suitability for larger objects’ recognition. However, small object features become less distinct and sometimes even blur in the background due to several downsampling operations. Therefore, a 160 × 160 feature map layer is added to facilitate accurate detection and localization of small bird targets in deeper feature maps. Consequently, an additional feature layer, P2, enriched with semantic information for small objects, is added to the neck of the model. The resulting model’s capability to retain the features relevant to small objects is improved by integrating superficial feature information into the deeper semantic layers that encode global information. Consequently, the model’s resolution and sensitivity to small targets are enhanced. Ultimately, the four recognition heads work together to help the model achieve a more comprehensive recognition performance.

#### 2.3.3. Improvement of Detection Head

As each detection head has independent feature inputs, there is a lack of information exchange between the heads, which deteriorates the detection performance. Additionally, the introduction of the P2 small-object-detection layer increases the number of floating-point operations in the model to some extent. To address these issues, we propose a lightweight, shared-detail-enhanced detection head (LSDECD), where group normalization (GN) is used to improve the classification and regression performance of the detection head, which enhances the detection efficiency [38]. Furthermore, detail-enhanced convolution (DEConv) [39] integrates traditional local descriptors into the convolutional layers through central difference convolution, angular difference convolution, horizontal difference convolution, and vertical difference convolution, which ensure accurate detection by strengthening the representation and generalization capabilities.

**Figure 6:**
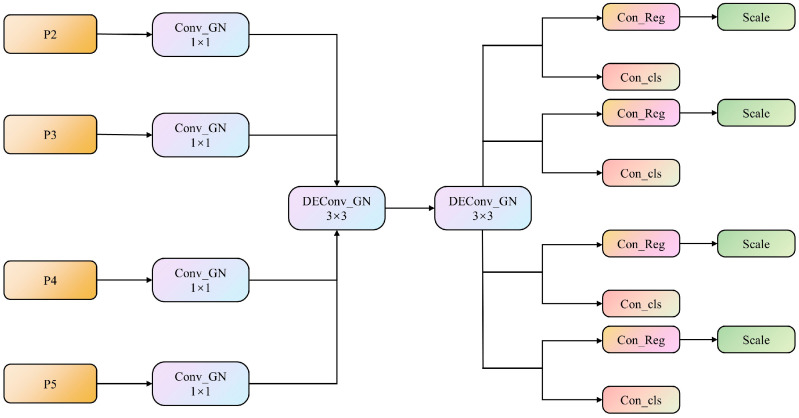
The overall structure of the lightweight, shared-detail-enhanced detection head. Conv_GN and DConv_GN denote the group-normalized convolution and group-normalized, detail-enhanced convolution, respectively. Con_Reg and Con_cls denote the convolutional modules for box regression and classification, respectively.

Once the feature inputs are received from the P2–P5 layers, the LSDECD first applies the GN-based standard convolution with a kernel size of 1 × 1 to each of the four levels. This step facilitates the information exchange across channels, enriches the amount of target information, and improves the classification and regression performance of the detection head. Second, two GN-based shared DEConv modules each with a kernel size of 3 × 3 are used to aggregate the rich spatial and semantic information from the four levels. Last, the information extracted by the shared convolution is input into the classification and regression heads (Figure 6). A scale layer containing a learnable scaling factor is employed to deal with inconsistent target sizes across detection heads and to prevent the loss of small-object feature information. This layer adjusts the features in the regression head, which enhances the multi-scale feature retention.

#### 2.3.4. Improvement of Loss Function

The scale of the targets changes drastically due to the frequent movement of birds, which makes it difficult for the model to precisely locate them. The original YOLOv8 network utilizes the Complete Intersection over Union (CIoU) loss function [40], which is primarily focused on the shape loss and exhibits a lower sensitivity to positional deviations in small objects. In contrast, the Shape-IoU loss is more effective at considering the inherent properties of bounding boxes, such as shape and scale, and their impact on regression. Thus, it can handle targets of varying shapes and sizes more effectively, which improves its detection performance for small objects. The Shape-IoU loss function is formulated as follows:

(5) $$IoU=\frac{\left|b\cap b^{gt}\right|}{\left|b\cup b^{gt}\right|}$$

(6) $$ww=\frac{2\times {\left(w^{gt}\right)}^{scale}}{{\left(w^{gt}\right)}^{scale}+{\left(h^{gt}\right)}^{scale}}$$

(7) $$hh=\frac{2\times {\left(h^{gt}\right)}^{scale}}{{\left(w^{gt}\right)}^{scale}+{\left(h^{gt}\right)}^{scale}}$$

(8) $$distance^{shape}=hh\times \frac{{\left(x_{c}-x_{c}^{gt}\right)}^{2}}{c^{2}}+ww\times \frac{{\left(y_{c}-y_{c}^{gt}\right)}^{2}}{c^{2}}$$

(9) $$\Omega^{shape}={\left(1-e^{-\frac{\left|w-w^{gt}\right|}{\max(w,w^{gt})}}\right)}^{\theta }+{\left(1-e^{-\frac{\left|h-h^{gt}\right|}{\max(h,h^{gt})}}\right)}^{\theta }$$

(10) $$L_{shape-IoU}=1-IoU+distance^{shape}+0.5\times \Omega^{shape}$$

In the above expressions, b and b^gt^ represent the predicted box and the ground truth box, respectively. The scale refers to the scaling factor, which is related to the size of the targets in the dataset. The width and height of the ground truth box are denoted as w^gt^ and h^gt^, respectively. The terms ww and hh represent the weight coefficients in the horizontal and vertical directions, respectively. Distance^shape^ represents distance loss. Ω^shape^ represents the shape cost. The value of θ defines how much the shape costs and is typically set to 4. L_shape-IoU_ represents the Shape-IoU’s bounding box regression loss. We improve the Shape-IoU based on the Inner-IoU^20^ by introducing a scaling factor to control the sizes of auxiliary bounding boxes. This factor further accelerates the convergence, balances the localization accuracy across targets of different scales, and improves the generalization performance. The Inner-IoU computation process is as follows:

(11) $$b_{l}^{gt}=x_{c}^{gt}-\frac{w^{gt}\times ratio}{2},b_{r}^{gt}=x_{c}^{gt}+\frac{w^{gt}\times ratio}{2}$$

(12) $$b_{t}^{gt}=y_{c}^{gt}-\frac{h^{gt}\times ratio}{2},b_{b}^{gt}=y_{c}^{gt}+\frac{h^{gt}\times ratio}{2}$$

(13) $$b_{l}=x_{c}-\frac{w\times ratio}{2},b_{r}=x_{c}^{gt}+\frac{w\times ratio}{2}$$

(14) $$b_{t}=y_{c}-\frac{h\times ratio}{2},b_{b}=y_{c}^{gt}+\frac{h\times ratio}{2}$$

(15) $$inter=\left(\min\left(b_{r}^{gt},b_{r}\right)-\max\left(b_{l}^{gt},b_{l}\right)\right)\times \left(\min\left(b_{b}^{gt},b_{b}\right)-\max\left(b_{t}^{gt},b_{t}\right)\right)$$

(16) $$union=\left(w^{gt}\times h^{gt}\right)\times {\left(ratio\right)}^{2}+\left(w\times h\right)\times {\left(ratio\right)}^{2}-inter$$

(17) $$IoU^{inner}=\frac{inter}{union}$$

Let b_l_, b_r_, b_t_, and b_b_ represent the left, right, top, and bottom boundaries of the predicted box, respectively. Furthermore, let $b_{l}^{gt}$, $b_{r}^{gt}$, $b_{t}^{gt}$, and $b_{b}^{gt}$ represent the left, right, top, and bottom boundaries of the ground truth box, respectively. The scaling factor, represented by a ratio, ranges from 0.5 to 1.5. The center point of the ground truth box and the inner ground truth box is represented by ($x_{c}^{gt}$, $y_{c}^{gt}$), while (x_c_, y_c_) represents the center point of the anchor and the inner anchor. Inter represents the area of the intersection between the predicted box and the ground truth box, while union represents the area of the non-overlapping portion between the predicted box and the ground truth box.

The integration of inner-IoU into the Shape-IoU framework to replace the IoU calculation component combines the advantages of both inner-IoU and Shape-IoU. This modification simultaneously accelerates the bounding box regression process and increases its precision, which allow the model to effectively capture the characteristics of birds and improve its generalization capability to deal with diverse bird species. The improved Inner-ShapeIoU is given by the following expression:

(18) $$L_{Inner-ShapeIoU}=L_{ShapeIoU}+IoU-IoU^{inner}$$

In the above expression, L_Inner-ShapeIoU_ represents the Inner-ShapeIoU’s bounding box regression loss.

### 2.4. Experimental Environment and Evaluation Metrics

#### 2.4.1. Experimental Environment

The experiments in this study were conducted on a Windows 10 64-bit operating system with an NVIDIA GeForce RTX 3090 GPU (NVIDIA Corporation, Santa Clara, CA, USA), powered by dual Intel (R) Xeon (R) Gold 5218 CPUs @ 2.30 GHz (Intel Corporation, Santa Clara, CA, USA), and 192 GB of RAM (IEIT Systems Co., Ltd., Jinan, China). The network model was implemented in the Python 3.10.9 environment using the PyTorch 2.0.1 deep learning framework.

#### 2.4.2. Evaluation Metrics

Multiple metrics were selected to comprehensively analyze the accuracy and real-time performance of the bird detection model. Precision and recall reflected the model’s ability to correctly identify targets and detect all relevant targets, respectively. Mean average precision (mAP) was used to assess the overall accuracy of the model. The computational complexity of the network model was quantified using GFLOPs, and parameters (Para) were used to evaluate the model size and complexity. Finally, frames per second (FPS) was employed to measure the real-time detection speed of the model.

The precision and recall were calculated as follows:

(19) $$Precision=\frac{TP}{TP+FP}$$

(20) $$Recall=\frac{TP}{TP+FN}$$

where TP, FP, and FN represent the numbers of correctly detected targets, falsely detected targets, and undetected targets, respectively.

The formula for mAP is given as

(21) $$mAP=\frac{1}{m}\sum_{i=}^{m}\int_{0}^{1}P(R)dR$$

where m denotes the number of target classes, P(R) represents the precision–recall curve, mAP@0.5 denotes the average AP of all categories when IoU is set to 0.5, and mAP@0.5:0.95 denotes the average AP of all categories at multiple IoU thresholds (ranging from 0.5 to 0.95, with a step size of 0.05).

## 3. Results

### 3.1. Module Improvement Experiment

The effectiveness of receptive field attention convolution was evaluated by comparing it with Alterable Kernel Convolution [41], Omni-Dimensional Dynamic Convolution [42], and Dynamic Convolution [43]. According to the experimental results (Table 1), RFAConv achieved the best detection accuracy, with a precision, recall, mAP@0.5, and mAP@0.5:0.95 of 94.2%, 87.5%, 93.2%, and 69.0%, respectively. Alterable Kernel Convolution can adjust the convolution kernel size based on the input to adapt to different target scales. However, it relies primarily on a fixed kernel structure when handling scale variations and lacks the ability to dynamically allocate receptive field weights. This limitation reduces its capability to accurately delineate target regions in scenarios with significant background noise. Omni-Dimensional Dynamic Convolution excels at capturing multi-dimensional contextual information but may struggle to distinguish between targets and the background. Dynamic Convolution dynamically generates convolution kernel weights to adapt to varying input features, making it suitable for general object detection tasks. However, it lacks targeted optimization for specific challenges, such as small-object detection. RFAConv’s ability to dynamically adjust receptive field weights based on the input context proves especially effective for handling variations in bird sizes and scales. Furthermore, it excels at suppressing background noise and focusing on target regions, enabling the extraction of fine-grained details. These advantages make RFAConv particularly well-suited to bird recognition in complex environments, where it outperforms the other convolutional modules.

The effectiveness of the integrated feature fusion module was evaluated through a series of comparative experiments that used the feature pyramid structure, which integrates Attentional Scale Sequence Fusion (ASF) as the baseline network. ASF only contains the SSFF module and TFE module. According to the experimental results (Table 2), when only the P2 small-object detection layer is used, the recall, mAP@0.5, and mAP@0.5:0.95 increase by 1.5%, 1.3%, and 1.3%, respectively, but the precision drops by 0.6%. When only the DySample is introduced, the evaluation metrics show minimal variations, with mAP improving slightly and precision and recall declining slightly. When the P2 small-object detection layer and DySample are introduced simultaneously, the recall, mAP@0.5, and mAP@0.5:0.95 increase significantly by 1.8%, 1.5%, and 1.3%, respectively, while only the precision decreases, by 0.2%. The introduction of the small-object detection layer significantly improves the small-object feature information extraction. However, the network’s precision is affected due to the insufficient acquisition of fine-grained details. The incorporation of dynamic upsampling improves the network’s ability to capture fine-grained features, mitigating the precision degradation caused by the P2 small-object detection layer. In summary, the reconstructed feature pyramid module DyASF-P2 demonstrates a superior detection performance.

### 3.2. Ablation Experiment

The effectiveness of the four improvements proposed in this paper was validated by comparing them to the baseline model, YOLOv8n. The experimental results (Table 3) show that each of the four enhancements improves the recognition performance, and they so to varying degrees. In individual module additions, when RFAConv is introduced, the receptive field attention mechanism allows the network to effectively capture target features, improving the backbone network’s feature extraction ability and reducing the impact of background noise. This increases the mAP@0.5 and mAP@0.5:0.95 by 0.4% and 1.1%, respectively. After introducing the DyASF-P2 feature fusion structure, the model captures rich fine-grained features of small objects, increasing mAP@0.5 and mAP@0.5:0.95 by 1.5% and 1.1%, respectively, and thus improving the detection accuracy for densely packed small targets. When LSDECD is added, mAP decreases slightly, with mAP@0.5 and mAP@0.5:0.95 dropping by 0.1% and 0.3%, respectively. However, this module optimizes the model’s parameters and floating-point operations (FLOPs), reducing them by 26% and 20%, respectively. The inclusion of Inner-ShapeIoU improves the algorithm’s ability to accurately locate multi-scale targets, consequently increasing mAP.

As far as the combination of multiple modules is concerned, the introduction of both RFAConv and DyASF-P2 further improves mAP compared to their individual contributions. This demonstrates that the four improvements play distinct roles in the recognition process: RFAConv effectively captures regions of interest, while DyASF-P2 enhances small-object detection. When LSDECD is subsequently added, mAP@0.5 increases by 0.2% and mAP@0.5:0.95 drops by 0.2%, signifying an overall stable accuracy but with a reduced model size and complexity. Finally, the addition of Inner-ShapeIoU further optimizes the already high recognition accuracy for multi-scale detection, and both mAP@0.5 and mAP@0.5:0.95 increase by 0.1%.

In summary, the experimental results demonstrate that YOLOv8n-bird has a significantly improved precision, with mAP@0.5 and mAP@0.5:0.95 increasing by 2.0% and 2.5%, respectively. The introduction of the small-object detection layer slightly increases the FLOPs: Compared to the baseline network, the FLOPs required by YOLOv8n-bird increase approximately by 4 × 10⁶. However, this increase in floating-point computation has a minimal impact on the performance in practical deployments given the overall balance of the model structure.

**Figure 7:**
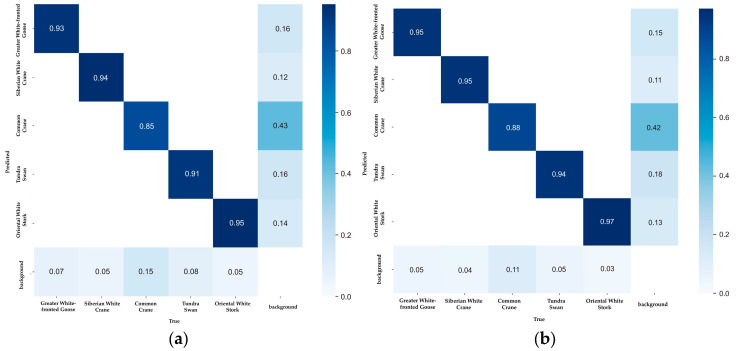
Comparison of the normalized confusion matrices: (**a**) YOLOv8; (**b**) YOLOv8-bird. In the confusion matrix, the elements on the main diagonal represent the number of correctly detected samples; the elements in the lower triangular region indicate the number of missed detections by the model; and the elements in the upper triangular region correspond to the number of false detections by the model.

The main diagonal elements of the YOLOv8n-bird confusion matrix have increased (Figure 7), indicating that the detection accuracy of these five bird species has been improved by YOLOv8n-bird. In addition, the values of the elements in the bottom row and the right column have decreased, which proves that YOLOv8n-bird can effectively distinguish targets from backgrounds and reduce false positives and false negatives of birds.

### 3.3. Comparison Test

The performance of the proposed YOLOv8n-bird model was compared with those of several other popular lightweight target detection models on the PYL-6-2023 dataset: RT-DETR, YOLOv3tiny, YOLOv5n, YOLOv6n, YOLOv8n, and YOLOv10. As the experimental results show (Table 4), YOLOv8-bird achievef the best detection accuracy, with a precision, recall, mAP@0.5, and mAP@0.5:0.95 of 94.6%, 89.4%, 94.8%, and 70.4%, respectively, while also having the lowest memory utilization of only 5.0 MB. In terms of real-time detection performance, YOLOv8n-bird reached 88 FPS, which was 30 FPS lower than the baseline model. The goal of this study was to develop a model with both high accuracy and fast detection capabilities, to meet the demands of real-time identification in video surveillance. YOLOv3tiny achieved the highest FPS at 172 FPS, which was 84 FPS higher than YOLOv8n-bird. However, it significantly underperformed in terms of precision, recall, mAP, and F1 score, making it the worst model in the comparison and considerably inferior to the YOLOv8n-bird model. Although YOLOv8n-bird achieved a lower FPS of 88, it is still sufficient to meet the requirements for mainstream FPS30 and FPS60 video surveillance, ensuring real-time detection capabilities. Overall, the YOLOv8n-bird model exhibited the most balanced performance, achieving high accuracy and low false detection rates while maintaining a lightweight design. This makes it ideal to address the challenges of multi-scale, dense small-object detection and achieve the real-time identification of rare bird species in the complex natural environments of Poyang Lake.

**Figure 8:**
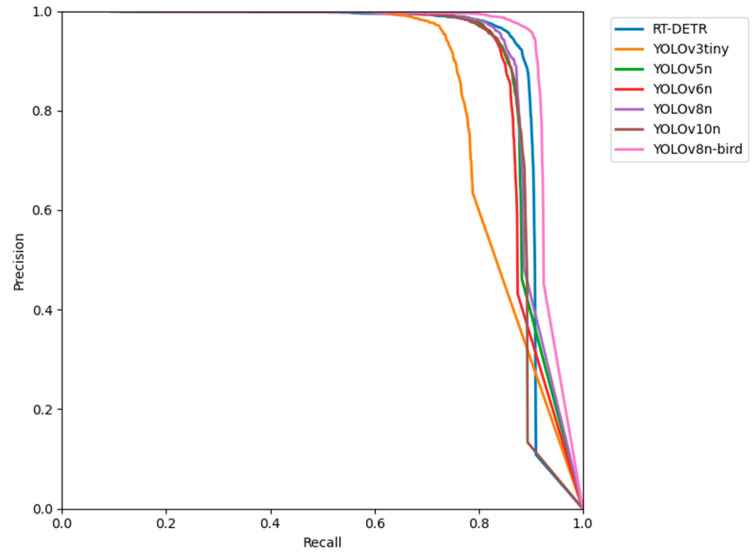
Precision–Recall curves. Precision–Recall curve is a graphical representation of a model’s precision and recall across different threshold settings. The The RT-DETR represents the Real-time Detection Transformer.

The Precision–Recall curve evaluates the performance of classifiers comprehensively using precision and recall as the key metrics. A curve closer to the top-right corner indicates that the model achieves higher precision and recall across different thresholds, reflecting a better overall performance. The YOLOv3tiny curve drops sharply at higher recall values (Figure 8), suggesting that the model experiences a significant decline in precision when detecting more targets, leading to an increased number of false positives. The overall performance levels of RT-DETR, YOLOv5n, YOLOv6n, YOLOv8n, and YOLOv10n are relatively similar, with strong performances observed in the medium-to-high recall regions. The YOLOv8n-bird curve lies above the other model curves, effectively enveloping them. This indicates that YOLOv8n-bird outperforms the other models in overall performance, further demonstrating the significant improvements achieved by the optimized YOLOv8-bird model in bird detection tasks.

### 3.4. Visualization

**Figure 9:**
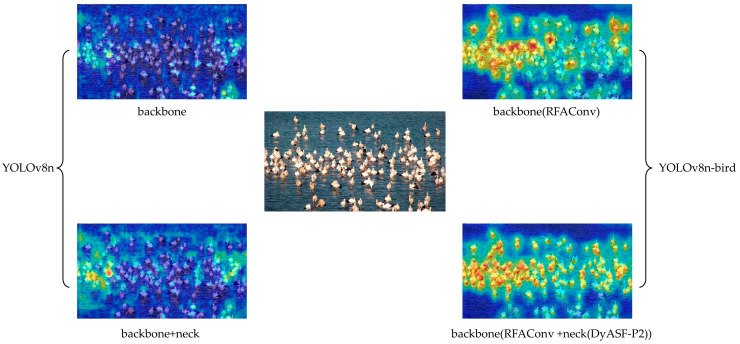
Comparison of YOLOv8 and YOLOv8-bird heat maps in each stage. The heatmap obtained from the YOLOv8 algorithm is on the left, the original image is in the middle, and the heatmap obtained by our proposed algorithm is on the right.

The effectiveness of YOLOv8-bird for object detection is demonstrated using Gradient-weighted Class Activation Mapping (Grad-CAM) heatmaps [44], which can be used to visualize images with dense occlusions. In these heatmaps, red areas indicate the regions of the network focus, with darker colors representing greater attention. YOLOv8′s performance is significantly affected by the background noise, due to which it cannot pay sufficient attention to the bird targets, and instead focuses only on a few high-density areas. This causes incomplete and non-specific feature extraction, leading to a higher rate of missed detections. In contrast, the heatmap obtained from YOLOv8-bird shows that the algorithm can effectively concentrate on the objects of interest, effectively distinguishing between birds and the surrounding environment. Its attention mechanism is broader and more distributed, and it almost covers all target areas (Figure 9). Additionally, the algorithm focuses on the key target features and pays greater attention to densely packed regions, which significantly enhances the bird detection performance.

**Figure 10:**
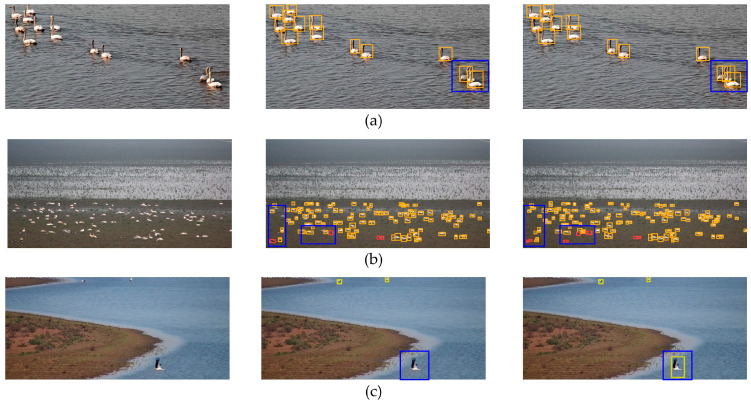
Comparison of YOLOv8 and YOLOv8-bird detection performance in each scenario: (**a**) occluded scene; (**b**) dense samll target scene; (**c**) multi-scale scene. The left image is the original, while the middle and right images show the detection results from the YOLOv8 and YOLOv8-bird models, respectively. The blue boxes highlight areas with a significant number of missed detections. The orange, red and yellow represent the detected Tundra Swan, Greater White-fronted Goose and Oriental White Stork.

We further compared the YOLOv8 and YOLOv8-bird algorithms’ bird detection performance levels, and the corresponding visualization results are shown in Figure 10. In occluded scenes, there are small swans with mostly white and gray light-colored feathers that lack clear contour boundaries when overlapped, which cause a detection failure with YOLOv8. However, YOLOv8-bird can still distinguish and identify the birds even under severe occlusion, thanks to its ability to capture fine-grained features. In scenes with blurred and densely packed small targets, YOLOv8 exhibits missed detection due to the small target size and the similarity between the birds and the soil color. On the other hand, YOLOv8-bird’s receptive field attention convolution allows for effective background noise suppression and enhanced feature extraction. Furthermore, the restructured feature fusion layer demonstrates strong representational capabilities, allowing the YOLOv8-bird model to identify some of the previously missed objects as it can capture rich feature details of small, blurred, and dense targets. In scenarios with large-scale variations, unlike the baseline network, YOLOv8-bird can detect large-scale flying Eastern White Storks thanks to its ability to capture multi-scale feature information. In conclusion, YOLOv8-bird can effectively detect birds in complex wetland environments and provides an efficient solution for accurately detecting densely occluded small bird species.

## 4. Discussion

In this study, we developed an improved object detection framework to automatically recognize birds in surveillance videos. The improved model achieved a mAP of 94.9%, which was 2.1% higher than that of the baseline model (YOLOv8). Due to the lack of standardized benchmark datasets, it is challenging to directly compare the accuracy of different deep learning models. Therefore, this discussion categorically focuses on the potential impact factors within the data quality and model improvement. Currently, commonly used bird image datasets are collected using unmanned aerial vehicles (UAVs), surveillance cameras, and trap cameras, resulting in varying data quality. UAVs as a flexible monitoring tool possess high mobility, enabling them to cover vast areas and transmit video data in real-time [45]. This makes UAVs particularly suitable for rapidly and dynamically monitoring bird populations during migration or other activities. However, UAVs also have limitations. First, bird images captured during flight are often taken from a distance, resulting in small bird sizes and insufficient detail [46]. This is especially problematic for smaller or more distant birds, potentially affecting recognition accuracy. Second, UAVs are constrained by limited battery life, which can hinder long-duration monitoring. Additionally, the noise generated during UAV flight may disrupt bird behavior, causing sensitive species to avoid the monitored area, thereby reducing monitoring effectiveness [47]. Trap cameras are commonly used for short-term monitoring at specific locations. They are triggered to capture photos or videos when birds pass by. Due to their relatively small monitoring range, trap cameras can focus on a bird at a specific site, making them suitable for observing individual birds or specific behaviors. For example, during the breeding season or along migration routes, trap cameras can capture high-quality images of individual birds. However, trap cameras’ narrow monitoring range and focus on individual birds make them less effective for monitoring groups of birds or recognizing targets in complex scenes [48]. Additionally, due to the reliance on triggering mechanisms, trap cameras may miss birds in certain periods or specific scenarios, and they lack a real-time monitoring capability. Surveillance cameras are typically deployed near bird habitats or migration routes to capture the full range of bird activity within the area. Their ability to operate continuously allows for the observation of bird behavioral patterns and habitat usage over extended periods. Unlike trap cameras and UAVs, surveillance cameras provide a broad coverage and can clearly record multiple birds simultaneously, making them particularly effective for detecting and counting bird groups. Due to their stability and extensive coverage, monitoring cameras are well-suited for long-term observation and multi-species monitoring in wetland environments. In term of model improvement, identifying bird species in complex environments presents significant challenges. On the one hand, existing network designs often focus on solving isolated problems [49,50,51], such as handling high-density scenarios or multi-scale variations. However, high-density detection often requires addressing occlusion, and detecting small targets frequently involves multi-scale issues. These challenges cannot be tackled in isolation, particularly in complex scenarios, where multiple factors, including background noise, must be considered comprehensively. As one of the most crucial wintering sites for migratory birds, Poyang Lake experiences a massive influx of birds by late November, necessitating solutions for high-density and occlusion challenges. Additionally, the varying distances of birds from surveillance cameras introduce the need for multi-scale recognition. On the other hand, current model improvements predominantly prioritize accuracy without considering the need for a lightweight design. While incorporating attention mechanisms [52] or Vision Transformers [53] can enhance precision, these additions increase computational complexity, requiring more resources and lowering efficiency. Real-time recognition tasks demand at least 30 FPS and, in some cases, up to 60 FPS, placing strict requirements on model optimization. The improved YOLOv8-bird model achieves an excellent balance between accuracy and efficiency, outperforming other models. It features a relatively small size, fast processing speed, and high classification accuracy, making it well-suited for deployment in surveillance cameras.

Although YOLOv8-bird has demonstrated significant advantages in bird detection, achieving true all-weather, all-time detection remains a challenge. Adverse weather conditions, such as heavy rain or fog, as well as low-light scenarios, can hinder accurate bird detection. Addressing these issues could involve incorporating a differentiable image-processing module, fog removal algorithms, or similar approaches to enhance network adaptability [54]. Another key factor is the quality and diversity of datasets. Bird detection, especially in complex environments like Poyang Lake, requires datasets with varied and extensive samples. Current datasets, constrained by manual labeling, cover only common bird species. Expanding these datasets to include more species across diverse weather, time, angle, and distance conditions is crucial. However, manual annotation is labor intensive and prone to human error, which can reduce model generalizability. Automated labeling techniques, combined with computer vision and deep learning algorithms, could improve the efficiency and accuracy of annotations. Data-augmentation techniques can also expand training datasets, enhancing model adaptability to different scenarios. Common supervised methods, such as rotation, translation, mirroring, and mixing, can improve robustness to varied perspectives. Unsupervised approaches, such as Generative Adversarial Networks [55] or AutoAugment [56], can generate new training samples based on existing data characteristics, further increasing diversity and quality [57]. Lastly, image resolution also significantly impacts bird detection. While training with high-resolution images can improve precision, particularly for small targets, it increases computational demands. For practicality, YOLOv8-bird was trained with a default resolution of 640 × 640, balancing computational load and accuracy. Image preprocessing was achieved by padding the shorter side of images and using bilinear interpolation for scaling. Bilinear interpolation calculates new pixel values as weighted averages of neighboring pixels, producing smooth, artifact-free images. However, it can blur details, particularly for small targets like bird feathers, and soften sharp edges, leading to detail loss. To address this issue, the network was optimized to expand the receptive field and enhance detail capture. From an image processing perspective, super-resolution reconstruction could be an effective solution. Image super-resolution techniques can restore details from low-resolution images, particularly for small targets, thereby improving recognition performance [58]. Deep-learning-based super-resolution methods, employing strategies like dense connections, memory mechanisms, and knowledge distillation, offer superior reconstruction results, further supporting bird detection tasks.

## 5. Conclusions

The bird images captured in natural scenes by video surveillance systems often exhibit significant scale and pose variations. Additionally, the detection of birds is frequently complicated by occlusion and background noise, which pose considerable challenges for conventional detection models. To address this issue, we proposed an improved lightweight detection method for real-time bird species monitoring from video surveillance data. The experimental results demonstrated that the proposed model significantly outperformed the baseline and other conventional object detection models in terms of precision, recall, and mAP. These results validate the model’s precision and efficiency in bird detection under complex environmental conditions, ensuring its applicability for real-time bird monitoring in surveillance systems. As more and more protected areas progressively establish comprehensive monitoring systems, vast amounts of video data are generated every day. The lightweight detection model can efficiently process these video data with limited computing resources, and it can be easily deployed on surveillance cameras to achieve the real-time detection of bird targets. This not only enhances the efficiency of bird monitoring systems but also broadens their potential applications. The application of this technology enables large-scale monitoring systems to operate efficiently in various environments. Based on these monitoring systems, combined with geographic visualization technology and web development technology, an intelligent real-time bird monitoring and recognition system can be developed. This system will enable the monitoring of large areas throughout the day and night, with a particular focus on endangered bird species, achieving minute-level monitoring. Such a system cannot only provide real-time feedback on the distribution and activity of birds but also provides important data support for bird conservation work. Our work can play an important role in the field of intelligent bird detection and monitoring, providing valuable technical support for bird conservation and biodiversity research. In the future, the scope of the research can be expanded to include bird tracking and flight trajectory prediction, further exploiting video data to conduct in-depth analyses of bird behavior and activity patterns. This will contribute to advancing bird conservation efforts by facilitating their greater precision and intelligence, while also aiding in wetland ecosystem protection.

## Figures and Tables

**Table 1:** Experimental results of different convolution modules.

Convolution Module	Precision (%)	Recall (%)	mAP@0.5/%	mAP@0.5:0.95/%
Receptive-Field Attention Convolution	94.2	87.5	93.2	69.0
Alterable Kernel Convolution	92.6	85.0	91.6	64.0
Omni-Dimensional Dynamic Convolution	93.2	85.7	92.3	65.8
Dynamic Convolution	94.1	86.9	92.9	68.1

**Table 2:** Experimental results of neck network structure improvement.

Improvement Section *		Precision (%)	Recall (%)	mAP@0.5 (%)	mAP@0.5:0.95 (%)
A	B				
×	×	94.5	87.1	92.8	67.7
√	×	93.9	88.6	94.1	69.0
×	√	94.4	86.8	93.0	68.0
√	√	94.3	88.9	94.3	69.0

Note: * In the Improvement Section, A and B represent the small-object layer and dynamic upsampling, respectively. √ means the module has been added. × means the module has not been added.

**Table 3:** The results of the YOLOv8-bird ablation experiment.

Improvement Section *				mAP@0.5 (%)	mAP@0.5:0.95 (%)	Parameters (×10^6^)	GFLOPs (×10^6^)
A	B	C	D				
×	×	×	×	92.8	67.9	3.1	8.1
√	×	×	×	93.2	69.0	3.1	8.6
×	√	×	×	94.3	69.0	2.5	12.0
×	×	√	×	92.7	67.6	2.3	6.5
×	×	×	√	92.9	68.1	3.1	8.1
√	√	×	×	94.6	70.6	2.6	12.6
√	√	√	×	94.8	70.4	2.2	12.1
√	√	√	√	94.9	70.5	2.2	12.1

Note: * In the Improvement Section, A, B, C, and D represent Receptive-Field Attention convolution, DyASF-P2 feature fusion network, lightweight, shared-detail-enhanced detection head, and Inner-ShapeIoU loss function, respectively. √ means the module has been added. × means the module has not been added.

**Table 4:** Comparison experiment between YOLOv8-bird and typical network models.

Model	Precision (%)	Recall (%)	mAP@0.5 (%)	mAP@0.5:0.95 (%)	FPS (f/s)
RT-DETR *	93.4	87.8	92.6	66.9	42
YOLOv3tiny	93.0	78.9	87.1	61.2	172
YOLOv5n	94.0	86.2	92.5	67.0	122
YOLOv6n	93.3	85.3	91.4	65.7	126
YOLOv8n	94.3	86.8	92.8	67.9	118
YOLOv10n	93.6	85.7	92.0	65.8	83
YOLOv8n-bird	94.7	89.9	94.9	70.5	88

Note: * The RT-DETR represents the Real-time Detection Transformer.

## Data Availability

The data presented in this study are available on request from the corresponding author.

## Supplementary Materials

The following supporting information can be downloaded at https://www.mdpi.com/article/10.3390/ani14233353/s1, Figure S1: PTZ camera; Figure S2: Detailed information on PYLB-5-2023; Figure S3: Overall structure of the triple feature-encoding module; Table S1: Detailed information about the PTZ camera.
