# Supplementary material for: An Improved Bird Detection Method Using Surveillance Videos from Poyang Lake Based on YOLOv8
Source: Animals (Basel). 2024 Nov 21;14(23):3353. doi: 10.3390/ani14233353 (PMC11639462; doi:10.3390/ani14233353)
Supplement: Supplementary file 1 [file animals-14-03353-s001.zip › animals-3294838-supplementary.pdf]

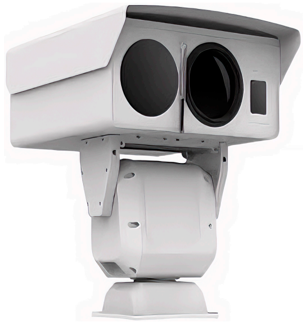

Figure S1. PTZ camera.

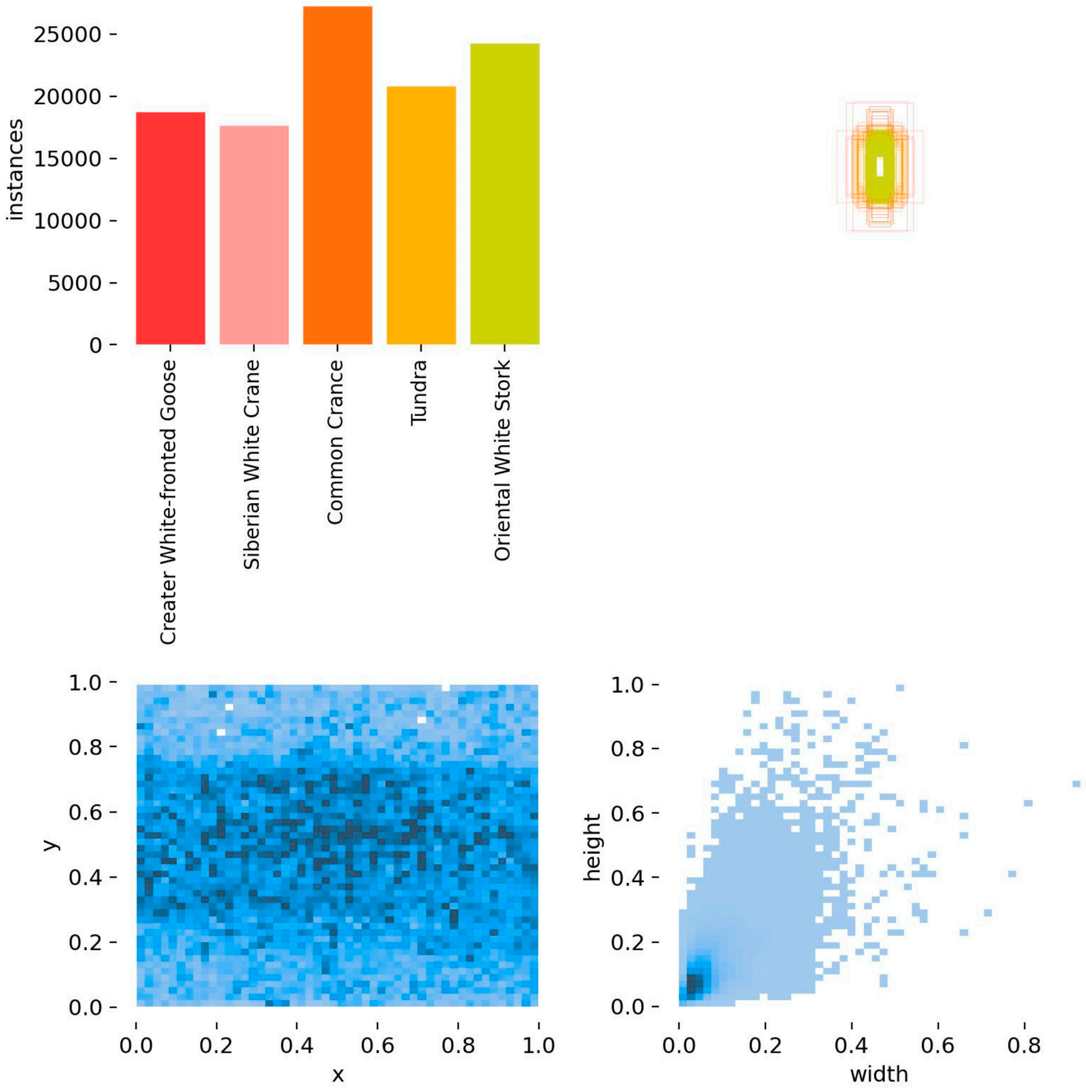

Figure S2. The detailed information about PYLB-5-2023.

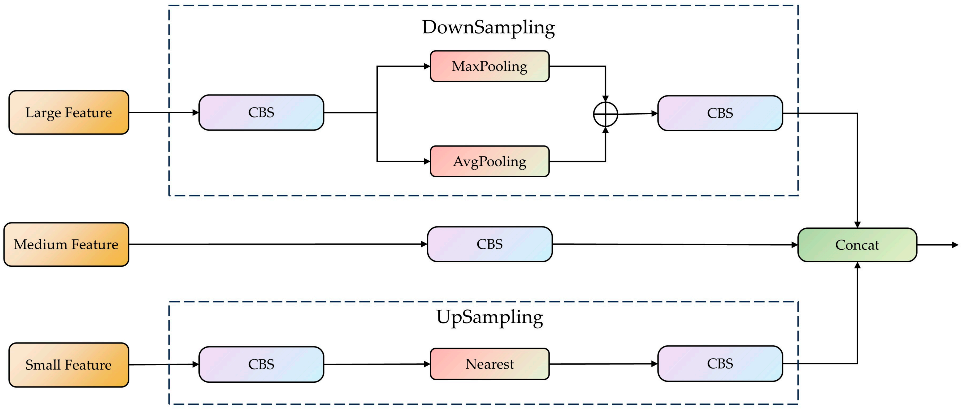

Figure S3. The overall structure of the Triple Feature Encoding module.

Table S1. The detailed information about PTZ camera.

Parameters	Value
Image Sensor	1/1.8" CMOS
Max. Resolution	1920 (H) × 1080 (V)
Min. Illumination	Color: 0.005 Lux @ (F1.2, AGCON)<br>B/W: 0.001 Lux @ (F1.2, AGCON)<br>0 Lux with laser
Focal Length	6 mm – 318 mm
Optical Zoom	53×
Pan/Tilt Range	Pan: 0° to 360° endless; Tilt: -45° to +45°
Manual Control Speed	Pan: 0.1°/s – 120°/s; Tilt: 0.1°/s – 50°/s
Preset Speed	Pan: 120°/s; Tilt: 50°/s
Main stream frame rate resolution	50Hz: 25fps (1920x1080)<br>60Hz: 30fps (1920x1080)
Storage	NAS (NFS, SMB/CIFS), ANR,<br>Micro SD card (256 GB)
GPS	Support
